# A Root Tip-Specific Expressing Anthocyanin Marker for Direct Identification of Transgenic Tissues by the Naked Eye in Symbiotic Studies

**DOI:** 10.3390/plants10030605

**Published:** 2021-03-23

**Authors:** Yiting Ruan, Ke Chen, Yangyang Su, Suyu Jiang, Ping Xu, Jeremy D. Murray

**Affiliations:** 1 CAS-JIC Center of Excellence for Plant and Microbial Science (CEPAMS), Center for Excellence in Molecular Plant Sciences (CEMPS), Shanghai Institute of Plant Physiology and Ecology (SIPPE), Chinese Academy of Sciences, Shanghai 200030, China; ruanyiting@cemps.ac.cn (Y.R.); jiangsuyu@cemps.ac.cn (S.J.); 2 Shanghai Engineering Research Center of Plant Germplasm Resource, College of Life Sciences, Shanghai Normal University, Shanghai 200234, China; keeechen@163.com (K.C.); suyangyang765@163.com (Y.S.)

**Keywords:** screening marker, *MtLAP1*, tissue-specific expression, hairy root transformation, root cap, *M. truncatula*, tomato

## Abstract

The *Agrobacterium rhizogenes* hairy root transformation system is widely used in symbiotic studies of model legumes. It typically relies on fluorescent reporters, such as DsRed, for identification of transgenic roots. The MtLAP1 transcription factor has been utilized as a reporter system in *Medicago truncatula* based on production of anthocyanin pigment. Here, we describe a version of this reporter driven by a root-cap specific promoter for direct observation of anthocyanin accumulation in root tips, which allows the identification of transgenic hairy roots by the naked eye. Results from our analysis suggest that the reporter had no significant effects on nodulation of *M. truncatula*. This approach, by virtue of its strong and specific expression in root cap cells, greatly reduces false positives and false negatives, and its use of an easily scored visible pigment should allow greater versatility and efficiency in root biology studies.

## 1. Introduction

The model legume species *Medicago truncatula* has been used extensively to study interactions with nitrogen-fixing rhizobia and arbuscular mycorrhizal fungi [1,2,3]. Both interactions occur within plant roots, and *M. truncatula* has also been used for studies on root biology, such as in hormone regulation, nutrient response, water stress, root architecture, etc. [4,5]. A common approach to study root biology at the molecular level in plants other than arabidopsis is to generate composite plants with transgenic roots using the *Agrobacterium rhizogenes*-based ‘hairy root’ transformation system. Hairy roots can be both nodulated and colonized by arbuscular mycorrhiza, and are thus very useful for symbiotic studies, particularly since transgenic roots can be obtained within several weeks while conventional regeneration of transgenic plants roots takes 3 to 6 months [6]. However, the efficiency of hairy root transformation is not 100%, and it is therefore essential to distinguish transgenic and non-transgenic roots. This is most commonly achieved using β-glucuronidase/GUS staining and fluorescent proteins (GFP, YFP, DsRed, etc.) encoded in plasmids being transformed, but these identification methods have many limitations [7,8]. For example, GUS staining assay is destructive, preventing further study of the material [9]. Observation of fluorescent markers needs a specially equipped microscope, and there may be problems with background autofluorescence depending on the plants or tissues being studied and fluorescent proteins being used. Another issue is that use of a fluorescent reporter protein often precludes its use in protein fusions, and so reduces the options available to the researcher.

In recent years, several studies have indicated the feasibility of using anthocyanins as a visible marker to avoid using expensive equipment and tedious histochemical procedures. Zhang et al. designed a set of binary transformation vectors (pPurpleRoot) using the *MtLAP1* (*Legume Anthocyanin production 1*) gene, and demonstrated its use for direct identification of transformed plants in *M. truncatula* [10]. Fan et al. also developed an anthocyanin reporter for several legume species including *Glycine max*, *Lotus japonicus*, *L. corniculatus*, and *M. truncatula* by using the *AtMyb75/PAP1* (*PRODUCTION OF ANTHOCYANIN PIGMENTS 1*) gene [11]. Both markers are suitable for non-destructive and direct visual identification of transgenic hairy roots.

*MtLAP1* is a MYB transcription factor that induces the production and accumulation of anthocyanin in legumes [12]. Anthocyanins are pigmented secondary metabolites with important physiological functions in plants like UV-shielding in leaves and coloration of flowers and fruits [13]. They are a type of flavonoid and share the same precursors, such as tetrahydroxychalcone and naringenin, as other important flavonoids like flavones, flavonols and isoflavonoids, which play essential roles in the nodulation process [14,15]. The accumulation of anthocyanins can be easily affected by sucrose and light treatments. It has been shown that flavonoid and anthocyanin biosynthetic pathways are strongly up-regulated following sucrose treatment while light signaling also induces anthocyanin biosynthesis via the AN3 and COP1 network [16,17]. Thus, these biochemical features of anthocyanins need to be taken into consideration when they are used as the transgenic reporter and the transformation system may also require optimization.

Here, we describe a new binary vector (pMtRC-MtLAP1) that carries a reporter gene, *MtLAP1*, under a root-cap specific promoter. Anthocyanin accumulation can be seen in the root tip of transgenic roots by the naked eye, which allows the easy visual screening of transgenic roots without the use of histochemical staining or fluorescent microscopy. In addition, we show that the *MtLAP1* marker does not affect *M. truncatula* nodulation. We also investigate the endogenous production of anthocyanins in roots responding to light and sucrose treatments and their effects on identification of transgenic roots and compare transformation efficiency between pMtRC-MtLAP1 and pAtUbi10-DsRed, which is the most commonly used marker for *M. truncatula* hairy root transformation.

## 2. Results

### 2.1. Anthocyanin Markers Allow the Easy Visual Screening of Transgenic Roots

**Figure 1:**
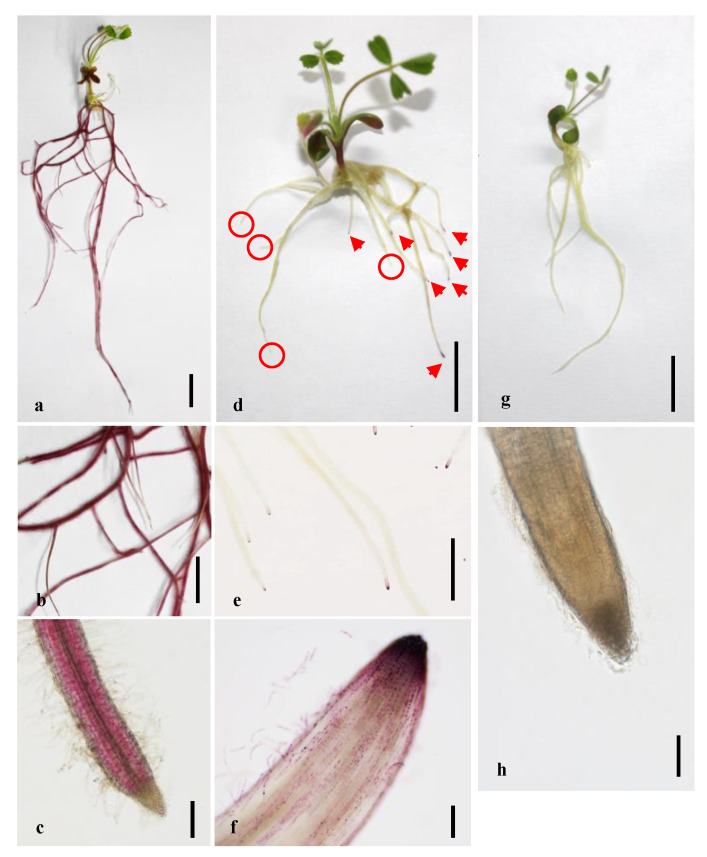
Anthocyanin markers allow the easy visual screening of transgenic roots in *M. truncatula*. (**a**–**c**) Purple coloration caused by anthocyanin accumulation can be observed in the root endodermis and pericycle after transformation with pAtE47-MtLAP1 (A17 plant grown on MFP medium for 18 days); (**d**–**f**) Strong coloration of the root tips (red arrows) observed after transformation with pMtRC-MtLAP1 allow transgenic roots to be easily distinguished from non-transgenic roots (circles) by the naked eye (A17 plant growing on MFP medium for 14 days); (**g**,**h**) No coloration was seen in the root tips after transformation with pAtUbi10-DsRed (A17 plant grown on MFP medium for 16 days). Bars are 10 mm (**a**,**d**,**g**), 5 mm (**b**,**e**), 200 µm (**c**,**f**), and 100 µm (**h**).

To confirm that the expression of *MtLAP1* in roots of *M. truncatula* can induce anthocyanin accumulation, an *AtE47* promoter-driven *MtLAP1* construct, named pAtE47-MtLAP1 (Figure S1a), was transformed into the *M. truncatula* ecotype A17 by *A. rhizogenes*-mediated hairy root transformation. As reported by Zhang et al., we observed roots with strong anthocyanin coloration (Figure 1a–c) [10]. The anthocyanins were deposited mainly at the endodermis and pericycle due to the specificity of the AtE47 promoter as previously reported (Figure 1c) [10].

To construct a binary vector using a root tip-specific expressing promoter to drive *MtLAP1*, we took advantage of the Golden Gate cloning system, which can easily assemble multiple DNA fragments in a desired order [18]. The resulted vector was named pMtRC-MtLAP1 (Figure S1b), which was transformed into *A. rhizogenes* strain ARqua1 for generating transgenic hairy roots of *M. truncatula* cv. Jemalong A17 and R108 plants. The anthocyanin specifically accumulated in the tips of transformed roots in both A17 and R108 plants (Figure 1d,f; data not shown for R108). Upon closer inspection, we observed that the anthocyanins accumulated specifically in vacuoles of root cap cells and border cells, often forming dark purple precipitates (Figure S2a). The marker was also scorable in older nodulated plants that were grown in substrate (Figure S2b). In contrast, anthocyanins were absent in the non-transgenic roots (Figure 1d). Plants transformed with the control vector pAtUbi10-DsRed did not accumulate anthocyanins in their root tips (Figure 1g,h). The marker was also functional in tomato (*Solanum lycopersicum*), although transformation frequencies were very low (Figure S3).

### 2.2. Light and Sucrose Induce Endogenous Anthocyanin Production in Roots but Do Not Affect Visual Screening of Transgenic Roots Expressing pMtRC-MtLAP1

**Figure 2:**
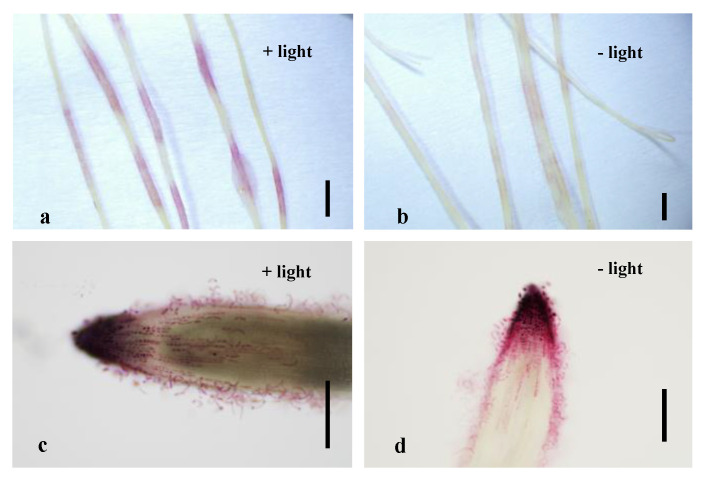
Light induces endogenous anthocyanin production in A17 roots but does not affect pMtRC-MtLAP1 visual screening of transgenic roots. (**a**) A17 roots showed purple sections in roots after transformation with pMtRC-MtLAP1 when grown under normal light conditions; (**b**) A17 roots showed few purple sections after transformation with pMtRC-MtLAP1 when grown under normal light conditions with roots shielded from light; (**c**) Anthocyanin pigments accumulate in root tips of A17 composite plants transformed with pMtRC-MtLAP1 with growth under normal light conditions; (**d**) Anthocyanin pigments in the A17 root tip after transformation with pMtRC-MtLAP1 with roots shielded from light. Plants were grown in an incubator on plates containing MFP medium. Bars are 1 mm (**a**,**b**), 150 µm (**c**), and 200 µm (**d**).

During our experiments, we occasionally observed purple sections along the roots transformed with either pAtUbi10-DsRed or pMtRC-MtLAP1 (Figure 2a). In order to determine whether it was caused by the transgenes and whether it could affect identification of transgenic roots expressing pMtRC-MtLAP1, we tested both transgenic and wild type A17 and R108 plants under different conditions. Sucrose treatment and light exposure are known to influence anthocyanin accumulation, so we tested their influence on the system. To limit light exposure, we wrapped the lower part of the plates with aluminum foil to cover the roots and shielded them from direct light, while others remained uncovered. To test the effect of sucrose, we added 10 g/L sucrose into the media. Interestingly, R108 plants did not develop any purple sections on roots of untransformed seedlings roots or transgenic roots expressing the pAtUbi10-DsRed in any of the treatments (Figure S4e–h; Figure S5e–h). However, in all A17 plants, the roots exposed to light exhibited clear purple sections, and sucrose and hairy root transformation enhanced the purple pigments on the roots (Figures S4a–d and S5a–d). Nonetheless, in both A17 and R108 plants, the root tips of all wild type and transgenic roots expressing pAtUbi10-DsRed under all treatments were white (Figures S4 and S5), while the ones expressing pMtRC-MtLAP1 were purple (Figure 2c,d). Based on this, we conclude that neither light treatment nor sucrose supplement affect the accumulation of anthocyanin pigments in the root tip of either genotype, although they both induce endogenous anthocyanin production in A17 root segments above the root tip.

### 2.3. The Apparent Transformation Efficiency of pMtRC-MtLAP1 Is Higher than pAtUbi10-DsRed

**Figure 3:**
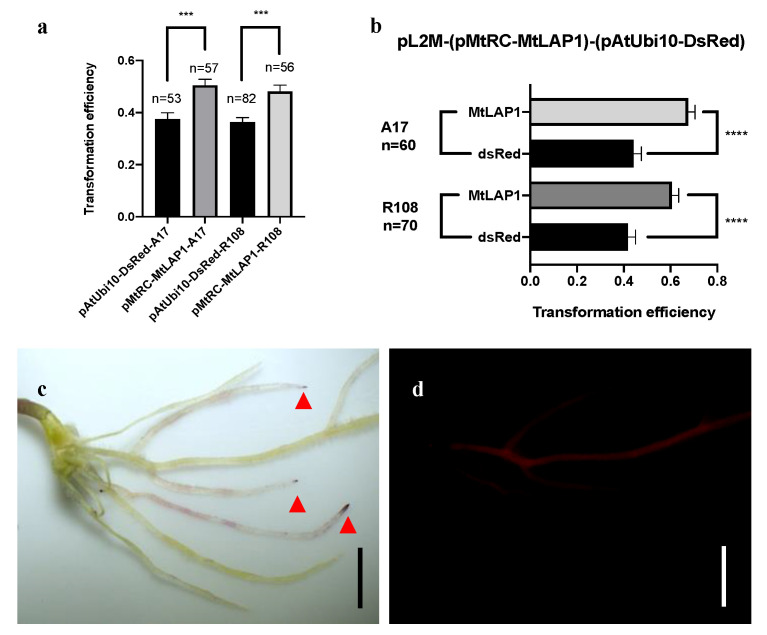
Transformation frequencies for pMtRC-MtLAP1 and pAtUbi10-DsRed on *M. truncatula*. (**a**) The frequency of plants having transformed roots using pAtUbi10-DsRed or pMtRC-MtLAP1; (**b**) The frequency of transformed roots per plant on those plants with at least one transgenic root with a vector containing both MtLAP1 and DsRed (pMtRC-MtLAP1 pAtUbi10-DsRed); (**c**) A17 transformed with pMtRC-MtLAP1 pAtUbi10-DsRed, red arrows indicate roots with purple tips that show either very weak or no DsRed fluorescence; (**d**) Same roots as in (**c**) under a fluorescent microscope. Bars are 2.5 mm in (**c**,**d**). Error bars in (**a**,**b**) are SEMs. Student’s *t*-test was used for statistical analyses, *** *p* < 0.001 and **** *p* < 0.0001.

After ensuring the feasibility of the pMtRC-MtLAP1 visual screening system, we investigated the transformation efficiency and compared it with the pAtUbi10-DsRed control vector. We then used these markers to test the percentage of plants that developed at least one transgenic hairy root. In both A17 and R108, over 50% of the plants (71–78% for A17, and 52–64% for R108) showed transformed hairy roots with either DsRed fluorescence or pMtRC-MtLAP1. Considering just the transformed plants, the frequency of transformed hairy roots for pAtUbi10-DsRed ranged from 36 to 38%, while for pMtRC-MtLAP1, it ranged from 48 to 51% (Figure 3a). We noted that DsRed-positive roots showed large fluctuations in intensity, which may result in false negative observations, while the pigment intensity of MtLAP1 positive roots was quite consistent. To test if the low transformation rates observed for DsRed were due to false negatives or were instead due to unforeseen effects of the transgenes on the transformation process, we constructed a vector that contained both markers (pAtUbi10-DsRed pMtRC-MtLAP1; Figure S1c). Upon transformation into *M. truncatula*, we found that almost every DsRed-positive root had a purple root tip, while many purple tipped roots did not show any DsRed fluorescence (Figure 3c,d). Thus, as observed when analyzing the markers independently, higher rates of transformation were estimated using the anthocyanin marker in both A17 and R108 backgrounds when the markers were present on the same vector (Figure 3b), confirming that pAtUbi10-DsRed produces false negatives. Together, these data indicate that pMtRC-MtLAP1 is a better reporter for transformation of *M. truncatula.*

### 2.4. The MtLAP1 Screening Marker Does Not Affect Nodulation

**Figure 4:**
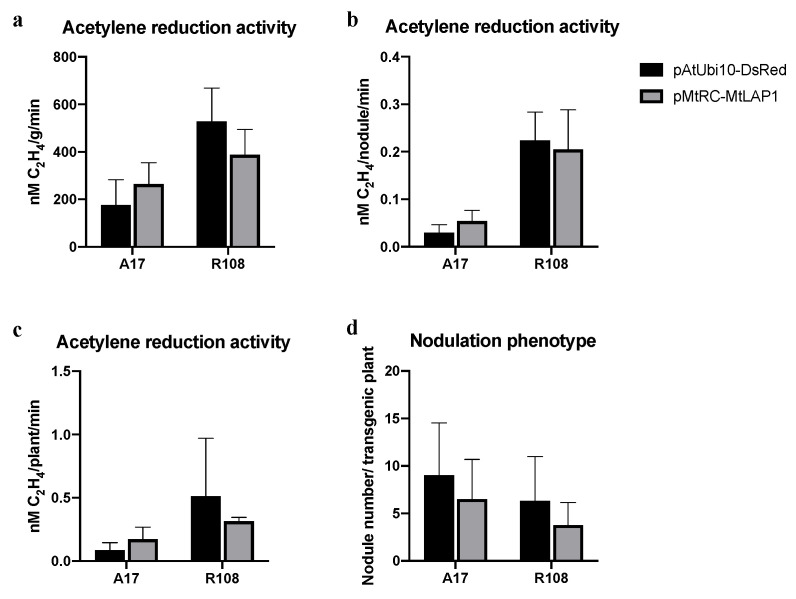
The MtLAP1 screening marker does not affect the acetylene reduction activity or nodule number of *M. truncatula* compared to DsRed. (**a**–**c**) Acetylene reduction activities 21 days after inoculation with *S. meliloti* 2011 presented on a per gram of nodule (**a**), per nodule (**b**) and per transgenic plant (**c**) basis; (**d**) Nodule numbers for plants transformed with pAtUbi10-DsRed and pMtRC-MtLAP1 21 days after inoculation with *S. meliloti* 2011 *HemA::LacZ.* Error bars are SDs. None of the means differed according to Student’s *t*-test (*p* < 0.05).

Hairy root transformation has been widely used for nodulation studies [19,20]. To test if the MtLAP1 reporter system is suitable for use in these studies, we assessed the number and nitrogen fixation potential of nodules in pMtRC-MtLAP1-transformed *M. truncatula* roots. The transgenic roots were inoculated with *Sinorhizobium meliloti* 2011 *HemA::LacZ*, and nodule number was calculated and the acetylene reduction assay was carried out 21 days later. Our results show that acetylene reduction activity and nodule number are not significantly affected in pMtRC-MtLAP1-transformed roots (Figure 4), suggesting that it is a useful marker for nodulation studies.

## 3. Discussion

In this study, we investigated whether anthocyanins produced exclusively in the root tip could be used as a screening marker for *M. truncatula* hairy root transformation. The *MtLAP1* expression was driven by the *MtRC* promoter, which is highly expressed in root tips. The expression of *MtLAP1* led to the accumulation of red/purple-colored anthocyanins in the root cap cells of *Medicago* and tomato, which can be seen without a microscope. The pMtRC-LAP1 marker was easily scored in both commonly used *Medicago* ecotypes, R108 and A17. Our analysis also showed that the effective transformation efficiency of the pMtRC-MtLAP marker compared favorably with that of DsRed, the latter being the most commonly used marker. We found this difference can be attributed to the large variation in DsRed expression in transgenic roots that results in a high rate of false negatives. Unlike DsRed, which is expected to have little or no impact on the cells in which it is expressed, MtLAP1 induces an entire suite of genes for production of anthocyanins, which could influence other metabolic pathways, for instance, flavonoid biosynthesis [12,21]. The anthocyanins could themselves impact on cell biology by blocking light or exerting effects as antioxidants [22,23]. Our use of a highly tissue-specific expression promoter helps decrease the risk of these effects on nodulation, and no obvious effects were observed on nodule numbers or N_2_-fixation capacity. Our results showed that using a sugar-free medium and shielding the roots from light can minimize anthocyanin production in roots for the A17 genotype, while neither sugar nor light-exposure induced anthocyanin accumulation in the R018 background. Importantly, neither sugar nor light exposure caused anthocyanin accumulation in roots tips of either genotype, and so, did not interfere with the pMtRC-MtLAP1 screening system. In conclusion, it is clear that anthocyanin production can be used as an effective marker for convenient visual screening for hairy root transformation to study the legume–rhizobia symbiosis.

This MtLAP1 anthocyanin marker, by replacing the common fluorescence indicator DsRed, makes DsRed or other fluorescent proteins with overlapping emission spectra available for use in protein fusions. The MtLAP1 system offers obvious advantages in efficiency over GUS staining. Another key advantage is the ‘live-staining’ aspect of the MtLAP1 system, allowing identification and monitoring of transgenic roots with minimal interference. We also successfully deployed pRC-MtLAP1 in hairy root transformation of tomato (Figure S3). Although its transformation efficiency was lower than *Medicago*, this might be improved by the use of different *Agrobacterium* strains. In addition, the anthocyanin accumulation appeared to be strictly limited to the tissues where *MtLAP1* was expressed, presumably due to glycosylation and vacuolization of the produced flavonoids [12]. This suggests that MtLAP1 can be developed as a marker to monitor gene expression in different tissues, particularly in the R108 background, a possibility that is currently being investigated.

## 4. Materials and Methods

### 4.1. Bacterial Strain, Plant Materials and Growth Conditions

*Escherichia coli* strain *DH5α* was transformed by heat shock and growth at 37 °C on LB (Luria-Bertani) agar (tryptone 10 g L^−1^; yeast extract 5 g L^−1^; NaCl 10 g L^−1^; 1.5% agar) plates containing relevant antibiotics for selection. *Agrobacterium rhizogenes* strain ArQua1 Ri (from Shanghai Weidi Biotechnology Co., Ltd., Shanghai, China) was grown in TY medium (tryptone 5 g L^−1^; yeast extract 3 g L^−1^; CaCl_2_ 0.5 g L^−1^; 1.5% agar) containing relevant antibiotics at 27 °C.

### 4.2. pAtE47, pMtRC, and pMtLAP1 Vector Construction

By using the MtGEA Differential Expression Analysis function in the NOBLE RESEARCH INSTITUTE (https://mtgea.noble.org/v3/), we identified Medtr4g059670, which we designate as *Root Cap* (*MtRC*). MtRC encodes a LEA protein (Affymetrix Probeset ID: Mtr.18366.1.S1_at) that shows tissue-specific expression strongly at the tip compared to that at the whole root (Figure S6). The promoter sequence of the *AtE47* (At2g37960) gene that shows tissue-specific expression in endodermis and pericycle in both *Arabidopsis thaliana* and *Lotus japonicus*, was amplified by PCR using wild type *A. thaliana* Columbia-0 genomic DNA as template by oligos pAtE47_F (5′-CTGTGGTCTCAGGAGGAAATTGGGCAGAAAATATG-3′) and pAtE47_R (5′–TTCGTGGTCTCACATTTATTTTTGCCTAATGAATGTTT-3′). The *MtLAP1* genomic sequence and promoter sequence of *MtRC* was amplified by using *M. truncatula* cv. Jemalong A17 genomic DNA as template by primers MtLAP1_F (5′-CTCTGTGGTCTCAAATGATGGAGAATACCGGAGGTGT-3′), MtLAP1_R (5′-TTCGTGGTCTCAAAGCTCAAGGTAGATCCCAAAGAG-3′), MtRC_F (5′ctgtggtctcaggagTTGAGTGTACAAATTCTGCAG) and MtRC_R (5′-TTCGTGGTCTCACATTCTTGGTGAACTCTCTTGATTT-3′) (homologous arm sequences are underlined). These fragments were then constructed into Level 0 vectors by seamless cloning, and *MtLAP1* gDNA was then assembled into a Level 1 vector in Golden Gate Cloning system with promoters *pAtE47* and *pMtRC*, to produce the vectors pL1M-pAtE47-MtLAP1-Adh and pL1M-pMtRC-MtLAP1-Adh. These level 1 vectors were used to construct Level 2 vector pL2M-(pMtRC-MtLAP1-Adh) -(pUbi-DsRed-Adh)-ELE2, which contains both *MtLAP1* and *DsRed*. These binary vectors were transformed into *A. rhizogenes* ARqua1 strain by electroporation.

### 4.3. A. Rhizogenes-Mediated Hairy Root Transformation

*M. truncatula* plants were germinated on water-agar plates at 22 °C in dark after being scarified with sandpaper, sterilized with bleach, washed with autoclaved dH_2_O, and imbibed for 3 h and vernalized at 4 °C in the dark for 4–7 days. An overnight liquid culture of *A. rhizogenes* strain ARqua1 containing the binary vector was grown. The seedling radicle was cut approximately 3 mm from the root tip after seedlings radicle length reached about 1 cm, and the wounded seedling was then dipped into the bacterial solution for about 10–15 min and then placed on a FP medium for 7 days at 22 °C (16 h photoperiod). The non-transgenic roots that grew before inoculation were then removed, and the seedling was transferred onto MFP medium. About 3 weeks after transformation, the number of plants with transgenic hairy roots was calculated for each treatment, plasmid construct, and plant genotype. The seedlings with at least one transgenic root were scored for transformation efficiency (percentage of transformed hairy roots on a given plant). The seedlings with transgenic roots were then moved into soil consisting of vermiculite and perlite (1:1) and grown in a greenhouse at 22 °C with 16/8 h light/dark cycle. One week later, the plants were inoculated with the rhizobia strain *S. meliloti* 2011 *HemA::LacZ* and nodulation phenotypes were scored 3 weeks after inoculation.

### 4.4. The Acetylene Reduction Assay

Nitrogenase not only catalyzes the reduction of atmospheric N_2_ to NH_3_ but can also reduce acetylene to ethylene, therefore reduction of ethylene by nitrogenase can be used to estimate N_2_-fixation capacity of nodules. For determination of acetylene-reduction activity (ARA), plants were either assayed 21 dpi with *S. meliloti* 2011 *HemA::LacZ* with tap water. For the AR assay, 3 to 7 biological replicates were used. Each replicate comprised of at least 7 nodulated roots for each genotype. The nodulated roots were introduced into 20 mL glass vials sealed with rubber stoppers. After injection of 1 mL acetylene, they were subsequently incubated for 2 h at room temperature. Samples of 200 μL of gas from each bottle were used to measure ethylene production using a gas chromatograph (7820A, Agilent Technology, Shanghai, China) and the roots were weighed. The nodules were then excised and weighed and the total nitrogenase activity was calculated as nanomoles of ethylene per gram of nodules, per nodule and per transgenic plant.

## Supplementary Materials

The following are available online at https://www.mdpi.com/2223-7747/10/3/605/s1, Figure S1: Diagrams of binary vectors; Figure S2: Anthocyanin accumulation in vacuoles and red root tips in nodulated plants; Figure S3: MtLAP1 system in tomato hairy root transformation system; Figure S4: Sucrose and light treatments on A17 and R108 wild types; Figure S5: Sucrose and light treatments on A17 and R108 transgenic plants transferred with pAtUbi10-DsRed; Figure S6: *MtRC* gene expression atlas in different tissues.
