# Supplementary material for: A Root Tip-Specific Expressing Anthocyanin Marker for Direct Identification of Transgenic Tissues by the Naked Eye in Symbiotic Studies
Source: Plants (Basel). 2021 Mar 23;10(3):605. doi: 10.3390/plants10030605 (PMC8004629; doi:10.3390/plants10030605)
Supplement: Supplementary file 1 [file plants-10-00605-s001.zip › plants-1153368-supplementary.pptx]

## Slide 1
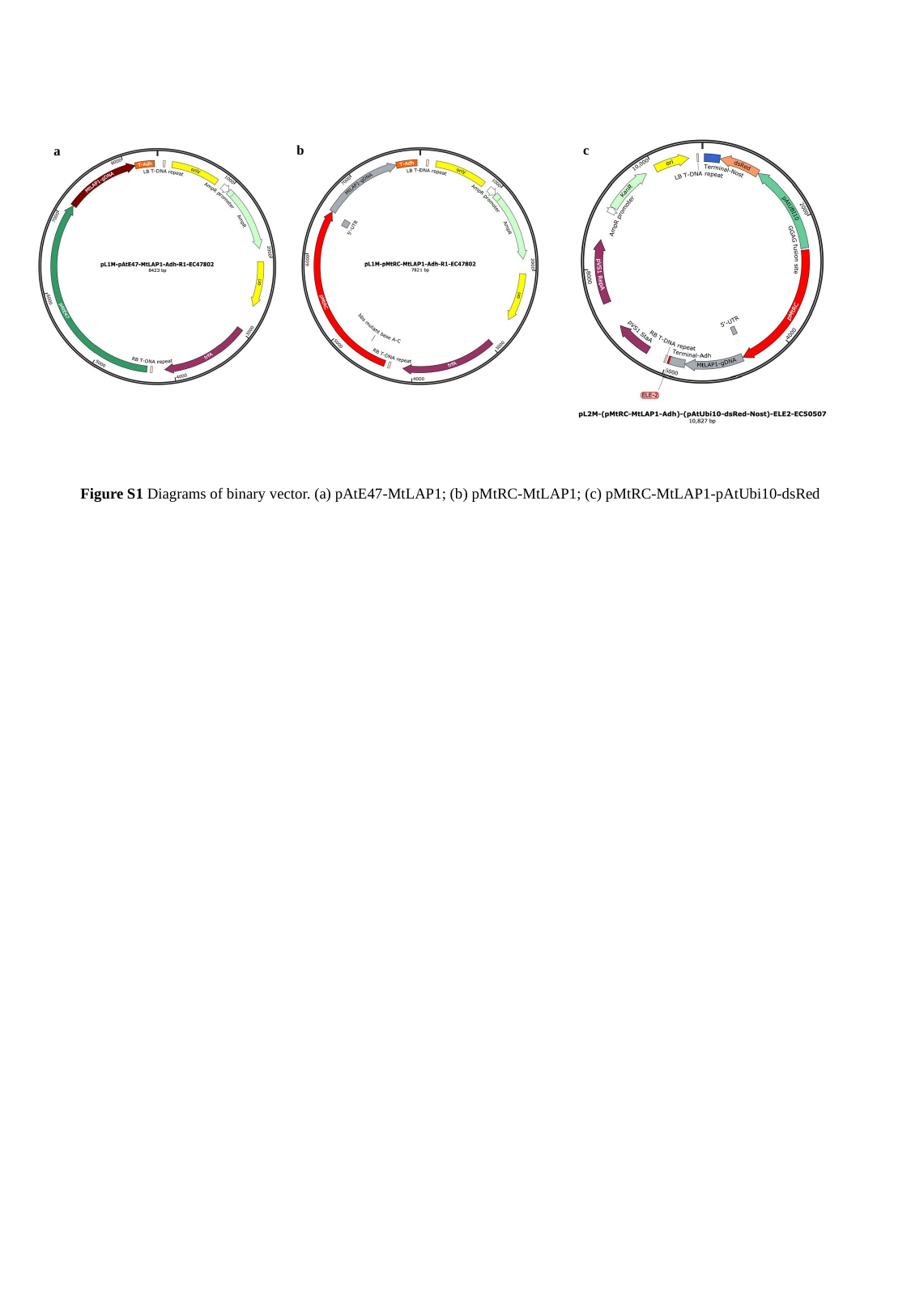

b
c
a
Figure S1 Diagrams of binary vector. (a) pAtE47-MtLAP1; (b) pMtRC-MtLAP1; (c) pMtRC-MtLAP1-pAtUbi10-dsRed

## Slide 2
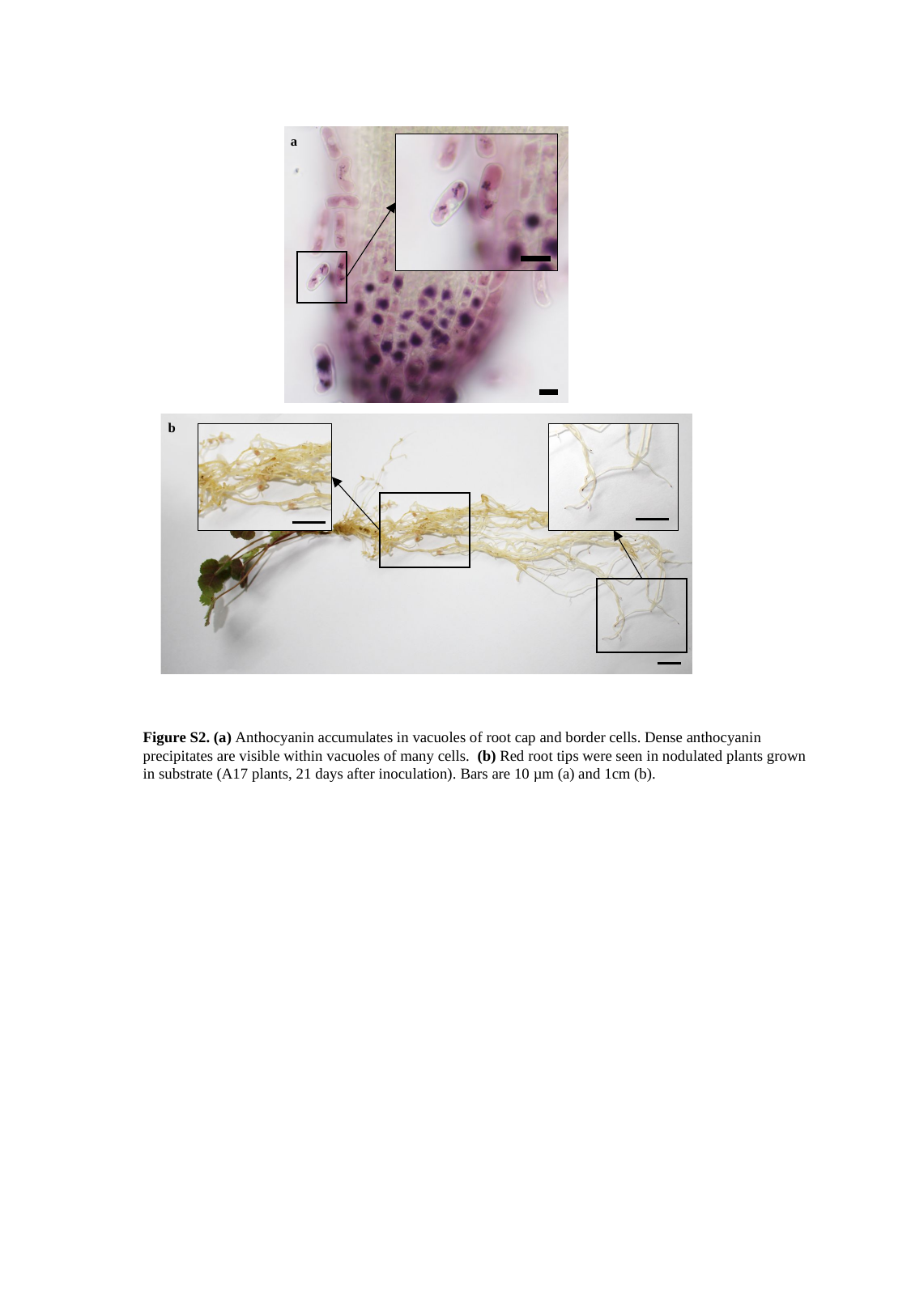

a
b
Figure S2. (a) Anthocyanin accumulates in vacuoles of root cap and border cells. Dense anthocyanin precipitates are visible within vacuoles of many cells. (b) Red root tips were seen in nodulated plants grown in substrate (A17 plants, 21 days after inoculation). Bars are 10 µm (a) and 1cm (b).

## Slide 3
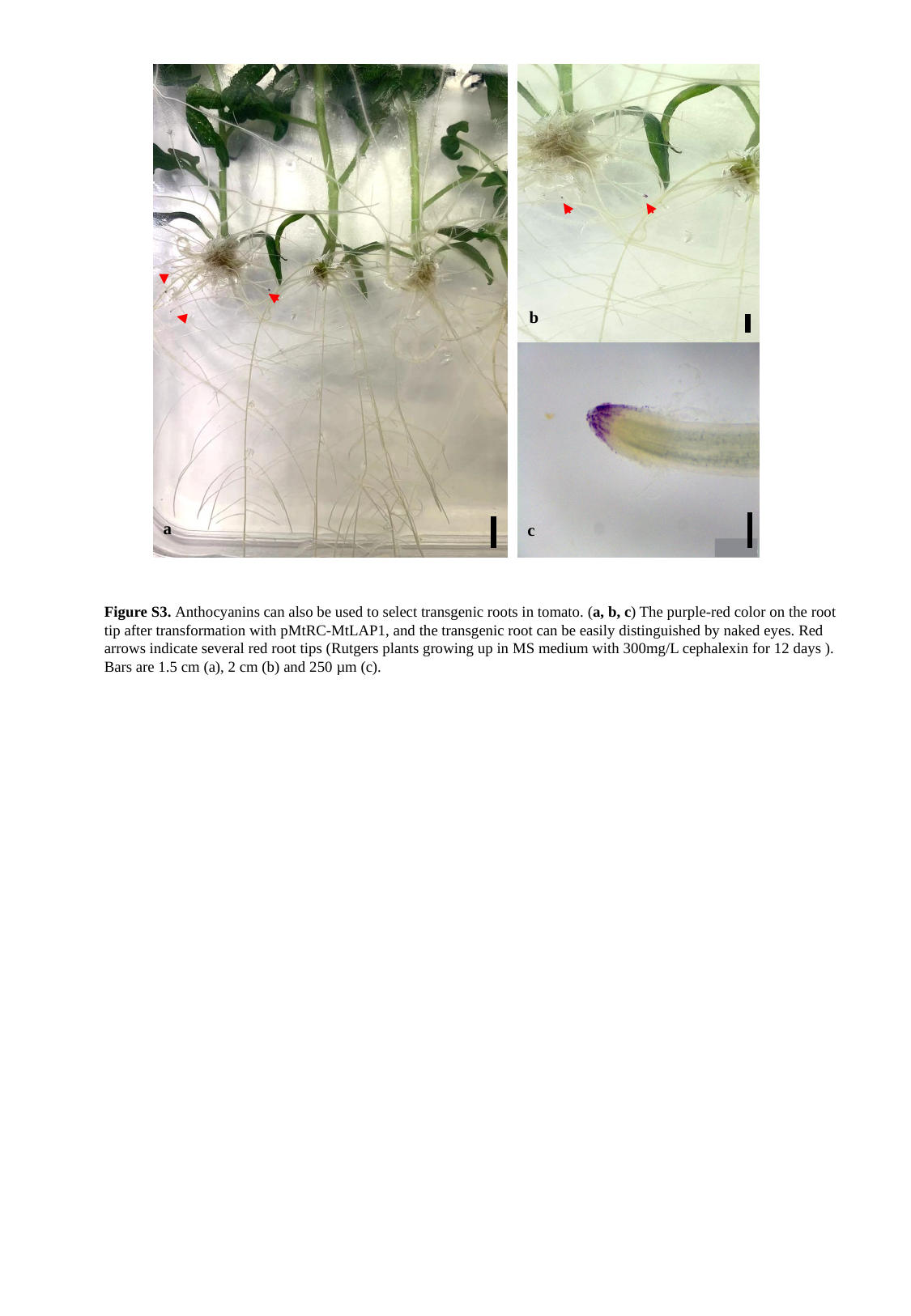

b
a
c
Figure S3. Anthocyanins can also be used to select transgenic roots in tomato. (a, b, c) The purple-red color on the root tip after transformation with pMtRC-MtLAP1, and the transgenic root can be easily distinguished by naked eyes. Red arrows indicate several red root tips (Rutgers plants growing up in MS medium with 300mg/L cephalexin for 12 days ). Bars are 1.5 cm (a), 2 cm (b) and 250 µm (c).

## Slide 4
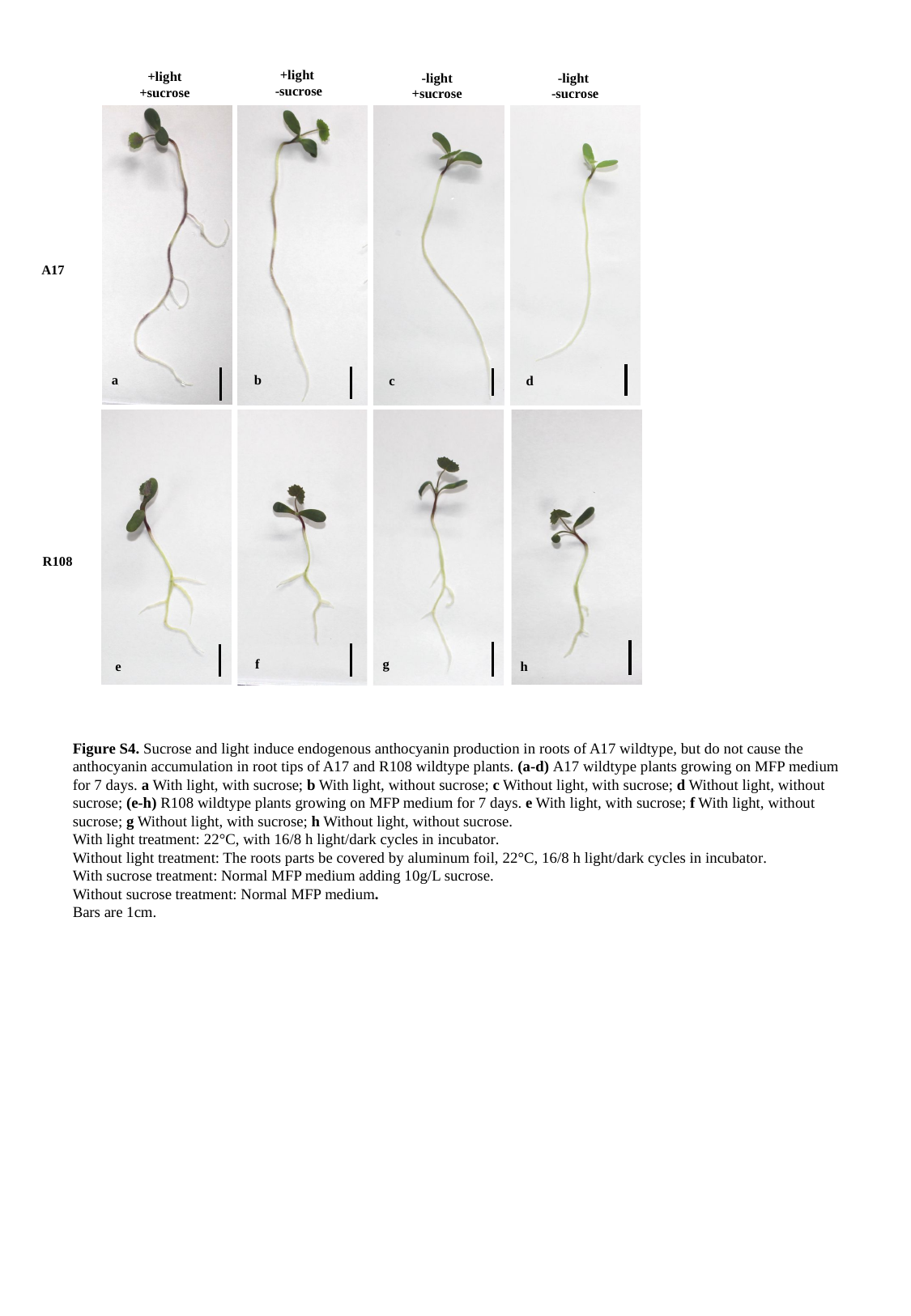

+light
-sucrose
+light +sucrose
-light +sucrose
-light
-sucrose
b
d
c
a
A17
f
g
e
h
R108
Figure S4. Sucrose and light induce endogenous anthocyanin production in roots of A17 wildtype, but do not cause the anthocyanin accumulation in root tips of A17 and R108 wildtype plants. (a-d) A17 wildtype plants growing on MFP medium for 7 days. a With light, with sucrose; b With light, without sucrose; c Without light, with sucrose; d Without light, without sucrose; (e-h) R108 wildtype plants growing on MFP medium for 7 days. e With light, with sucrose; f With light, without sucrose; g Without light, with sucrose; h Without light, without sucrose.
With light treatment: 22°C, with 16/8 h light/dark cycles in incubator.
Without light treatment: The roots parts be covered by aluminum foil, 22°C, 16/8 h light/dark cycles in incubator.
With sucrose treatment: Normal MFP medium adding 10g/L sucrose.
Without sucrose treatment: Normal MFP medium.
Bars are 1cm.

## Slide 5
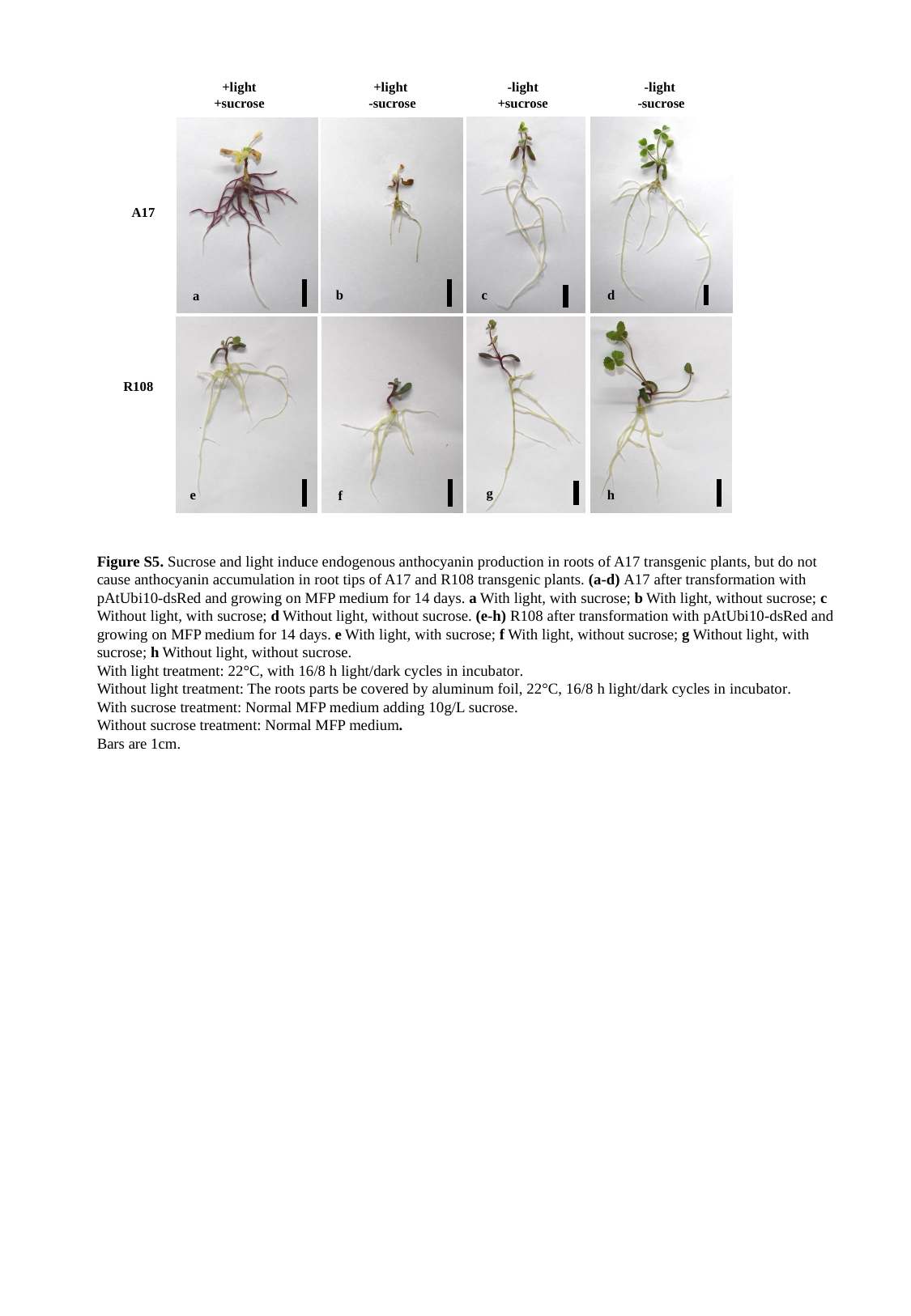

+light +sucrose
+light
-sucrose
-light +sucrose
-light
-sucrose
c
d
a
b
A17
e
f
h
g
R108
Figure S5. Sucrose and light induce endogenous anthocyanin production in roots of A17 transgenic plants, but do not cause anthocyanin accumulation in root tips of A17 and R108 transgenic plants. (a-d) A17 after transformation with pAtUbi10-dsRed and growing on MFP medium for 14 days. a With light, with sucrose; b With light, without sucrose; c Without light, with sucrose; d Without light, without sucrose. (e-h) R108 after transformation with pAtUbi10-dsRed and growing on MFP medium for 14 days. e With light, with sucrose; f With light, without sucrose; g Without light, with sucrose; h Without light, without sucrose.
With light treatment: 22°C, with 16/8 h light/dark cycles in incubator.
Without light treatment: The roots parts be covered by aluminum foil, 22°C, 16/8 h light/dark cycles in incubator.
With sucrose treatment: Normal MFP medium adding 10g/L sucrose.
Without sucrose treatment: Normal MFP medium.
Bars are 1cm.

## Slide 6
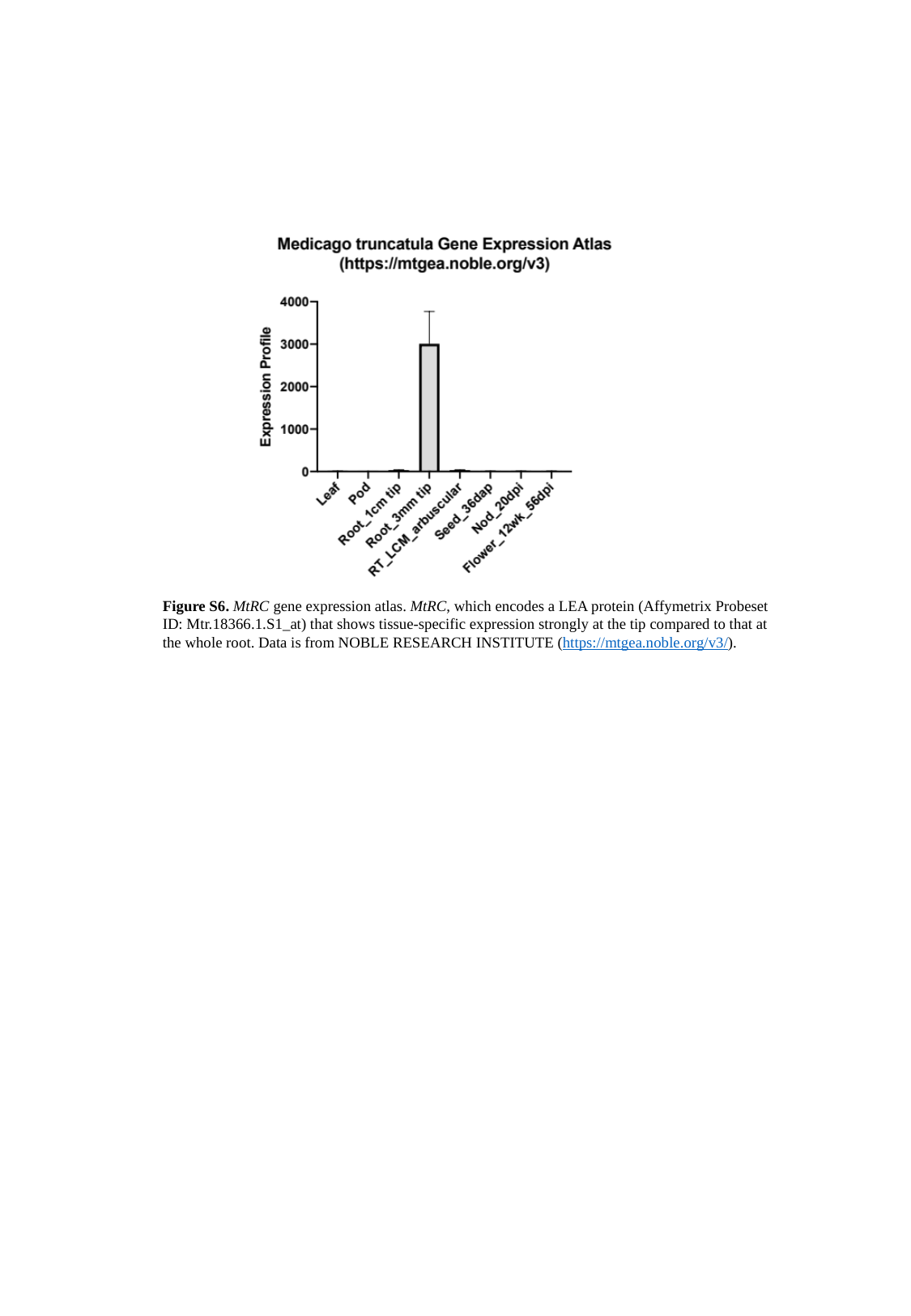

Figure S6. MtRC gene expression atlas. MtRC, which encodes a LEA protein (Affymetrix Probeset ID: Mtr.18366.1.S1_at) that shows tissue-specific expression strongly at the tip compared to that at the whole root. Data is from NOBLE RESEARCH INSTITUTE (https://mtgea.noble.org/v3/).
